# Artificial Local Anemia in Surgery*Translated from *Centralblatt fur Chirurgie*, August, 1896.—Ed. (pro tern.)

**Published:** 1896-11

**Authors:** F. Von Esmarch


### ARTIFICIAL LOCAL ANEMIA IN SURGERY.*

BY F. VON ESMARCH.

F. von Esmarch, the distinguished surgeon and discoverer of "bloodless operations," in an address on this subject made on the occasion of the 25th Jubilee Congress of the German Society of Surgery, held at Leipzig, May 27-30, 1896, said:

GENTLEMEN:—Our honored president has requested me, as senior member of our society, to deliver the initial address of this convention, suggesting at the same time that the subject of such address should be "Local Anemia."

Since I have upon different occasions, both in your presence and before other conventions, dealt with this subject, and moreover not having as a matter of fact much of a new nature to disclose, I shall confine myself, in order to infuse some interest into my attempt, to

Translated from *Centralblatt fur Chirurgie*, August, 1896.—ED. (pro tem.)

the task of handling it in perhaps a more able manner than heretofore.

I had the privilege, some twenty-three years ago, of communicating my discovery to you, but I did not then state the circumstances which led to its possibility, so that if you will permit me to refer to the past, you may not consider it a trespass on your attention if I now relate such circumstances to you.

By many persons, we surgeons are considered as being gruesome, bloodthirsty, and unfeeling. This is in direct contradiction of our nature. I shall assuredly be in sympathy with your feelings when I assert that a good surgeon must possess a compassionate disposition, and that it is precisely on account of our being thus constituted that we select a profession calling for the noblest instincts of humanity. What is it that stimulates our untiring energies oft into far declining age, if it is not the desire to alleviate the sufferings of our fellow men? If love for our neighbors is the first and foremost principle of the laws of Christianity, then verily we surgeons are better Christians than many of those who profess to call themselves such. I am one among you who accepted the responsibilities of our profession, and I have felt a profound satisfaction in its humane privileges.

But there have always been three crying evils which embittered the exercise of our calling. These are:

First. The pain occasioned to the patients by operation.

Second. The danger to which lives are exposed (infection) by operations.

Third. The *loss* of blood which unnecessarily follows.

I am happy I have lived to see these three evil conditions, if not entirely, yet in a high degree, removed, and that I have been able to assist here and there in combating them.

Some fifty years ago, as a student, I had witnessed many a bloody operation in Göttingen and Kiel; had even, as Bernhard Langenbeck's assistant, given practical aid to the same, when in the year 1846 there came from America the joyful tidings of Morton's discovery of the properties of ether as a narcotic.

Who has not lived to see it, can in no way conceive the exultation which then seized the doctors, and more especially the students in all surgical clinics.

Where heretofore the operating-rooms had been filled with screams and moans of the unfortunate patients, by the impress of which many a novice had been brought to a state of unconsciousness, now suddenly all was still, even deathly still, save interruptions only occasioned by wandering mutterings, even some times by joyful songs proceeding from the subject.

The discovery made a rapid tour of triumph over the entire globe, and was as quickly followed in 1847 by a new one, that of Simpson, which proved that chloroform had a more rapid effect in producing *narcosis* than ether. Thus it quickly superseded the latter. During the first Schleswig-Holstein war chloroform was introduced in Langenbeck's clinic, where its priceless value was fully realized and appreciated. Since then it was for some years extensively used in the clinic of Kiel until ether again came into use, and has since retained its sway.

Not so easy was the victory obtained in the field of infectious wounds, which threatened the hurt in battle and those operated upon and made the name of "Lister" immortal; and where is the surgeon to be found who is not especially grateful for the ever continuously increasing benefits that are derived from asepsis? benefits which Lister prophetically proclaimed in these words: "The treatment of wounds had the object of hindering the development of decomposition in a diseased part. When the fulfillment of this problem shall have been reached, then shall, indeed, surgery be viewed in a different light from heretofore. Diseases and wounds looked upon in a despairing, nay hopeless, light, will, on the other hand, have a steady and assured convalescence, and thus this new observation will become the polar star, whose light will guide our vessel over the desolate sea."

The third crying evil which has in all times attacked the progress of science and increased its difficulties was the unnecessary loss of blood. The diminution of such during operations has always been regarded by surgeons as of the utmost importance. Otto Weber, in his classical work on Histology of Diseases, refers to the treatment of hemorrhages, especially the stay of the flow of blood in an open wound, as being the root of surgery. The history of blood-staunching, he says, thus becomes the history of our science and may be adopted as the record of its progress or retrogression.

From the commencement of my surgical activity, during the many operations I have performed, this question has always been foremost in my thoughts, and as Langenbeck's assistant, I had many opportunities to strengthen my constant interest. Langenbeck had always twelve artery forceps prepared to seize each spouting vessel immediately upon cutting, and placed especial value on well waxed silk thread, as also the rapid, tight knotting of the same. As his assistant I have spent many a night in preparing the thread to be used the following day, for at that period we did not have the aid of benevolently disposed sisters.

When the first Schleswig-Holstein war broke out, I lost my advantages as assistant to my early instructor, having been made a prisoner of war during the first engagement. But when a cessation of hostilities was called, Langenbeck being summoned to Berlin, Stromeyer was appointed his successor as staff surgeon-general of the Schleswig-Holstein army. I then resumed my opportunities to put my knowledge to practical use, for Stromeyer frequently selected us young doctors (Langenbeck's pupils) to perform operations, and it became my duty to bind the bleeding arteries. Since we often found ourselves with an insufficient supply of waxed silk thread, I appealed to the ladies of the Central Aid Society in Kiel, who torthwith provided us with large quantities, and thus prepared the way for an unlimited supply furnished to our army surgeons in the following campaigns, till in the war of 1870–1 catgut superseded thread.

When later on in 1854, I became the head of the surgical clinic, I increased the number of artery forceps to forty, for which I frequently found use in important operations.

I was led to this step by a case that came under my notice when operating in 1852, upon a man of sixty-one years, who had a lipoma, a fatty tumor, weighing fifty-six pounds, whose broad base hung from the nape of his neck, the weight of which became unbearable.

With youthful ardor I applied myself to the task of its removal, but found myself confronted by several serious obstacles. Immediately following the first incision of the knife came a copious flow of blood which brought our twenty forceps into requisition and necessitated first of all the binding of all the vessels. But during this time the strength of my assistant gave out and two others were called to assist him in raising the weight of the tumor. Then suddenly I heard it said, "The pulse is gone; the patient is no longer breathing!" Chloroform asphyxiation had taken place, as the patient had to be laid on his stomach, so that it was only after much labor that we turned him on his back and by artificial respiration brought him back to life.

It was not until this had been accomplished that I was able to resume the operation, which I continued by separating the heavy mass of the tumor completely from the body by a rapid cut, using the whole of my forceps again and again, whilst my assistants were stemming the flow of blood with broad sponges. After rapidly inserting a few stitches, we finally succeeded in placing the man on his bed, but he never recovered himself again. Rigors ensued, and he succumbed to "pyemia" in a few days.

Had I to perform such an operation to-day the patient would lose but a small quantity of blood. In fact, Woefler, in 1878, amputated a tumor of the neck weighing fifty pounds with the most satisfactory results by means of "local anemia."

. . . . . . . . . . .

The most important operation I performed was that on a young girl on whose thigh a tumor of immense proportions, "elephantiasis teleangiectodes," had grown. Experience had taught me that those veins and arteries entering into the tumor may be greatly expanded or widened. I therefore experimented in the following manner in order to minimize the loss of blood:

I had two wooden splints made, provided at equal distances with holes. Having attached these firmly to either side of the base, I introduced in these holes a strong silver wire and drew them through by aid of long needles in an oblique direction, two of which I twisted together. The overlapping portions of the thigh I then bound firmly with iron wire. Proceeding then to cut the great tumor above the splints, I made a long wound of forty centimeters, and closed it by a number of deep and superficial stitches of silver suture. Only a small quantity of blood was lost during this long operation.

You will have observed by what I have said that I have been on several occasions in close contact with my discovery of local anemia, but the thought itself only really ripened in February, 1873, and in the following manner:

I was requested one evening by a lady to withdraw her wedding ring from her finger, which owing to a contusion had suffered a severe swelling. I have no doubt in my mind but that you are all acquainted with the course to pursue in such cases.

It is usual to bind the finger tightly from the point to the ring with strong thread, passing the end under the ring and then removing the thread by a quick effort whereby the ring passes easily over the shrunken finger to the point.

The lady, much pleased by the result, begged me to explain the process to her.

Intimating how, during the process of binding, the finger had first become white, then red, I easily explained the cause, namely, that the blood had been driven out of her finger by the pressure of the binding process, and that consequently the dimensions of her finger had decreased considerably.

After she had left my thoughts dwelt for a long time on this process, amusing myself the while by binding my finger first with thread, then with thin rubber thread in different directions, observing that by means of the cotton each subsequent trial occasioned a more unpleasant effect. I dreamt of it all that night. Awaking the following morning, suddenly the ripe thought appeared to my internal vision: *From now on you must before every operation press the blood out of the limb, and not permit it to enter again until the operation is completed.*

Then my thoughts turned to the future, and I pictured the many difficult cases where my discovery would be brought to bear as a blessing to mankind.

Naturally I felt very happy. On the same day I removed a pieec of collar bone, and on the following did an "exarticulation" of the hand, as also several scrapings of carious bones—all these without loss of blood—and was each time surprised how much easier these operations became under treatment suggested by my discovery than before.

I was also most anxious to communicate my discovery to my colleagues, in order that its benefits might reach mankind as speedily as possible. The holidays were at hand, and with them the second surgical congress, where I delivered my first address on the production of local anemia.

It was my own fault if I did not succeed in making any good impression, for I neglected to prove my subject by living illustrations or intelligible plates, but I felt discouraged, nevertheless, when I found that no attention was paid to it subsequently by any of my colleagues, a proof that the thought had not excited a corresponding enthusiasm, as Stromeyer said. However, some consolation I found in the hope that perhaps one or the other would eventually try the experiment and understand the worth of its knowledge.

In the following summer I performed a number of operations with exceptional results, among others, along with my then assistant, the present Prof. Peterson, the removal of two sequestra on both tibiæ without any loss of blood. These I subsequently illustrated in Volkmann's clinical addresses, No. 58.

During the following autumn the army surgeons' exhibition was held in Vienna, when I amputated two thighs and made a resection with artificial local anemia in the presence of surgeons of all countries, who then carried the tidings of the discovery to their homes.

In the preface of his classical work on "Neurasthenia," George Beard very correctly says: "Every new thought penetrating into the world passes through three periods before being fully and unquestionably accepted by the profession:

1. The period of indifference.
2. The period of negation.
3. The period of priority."

My discovery passed through the first period quickly, for already in the course of the same year many eminent surgeons (Billroth, Langenbeck, Bardeleben, Brandis, Albert, Leisrenk, MacCormac, Simon, Stokes, etc.,) raised their voices in its favor, discussing its advantages with more or less enthusiasm.

To this period soon succeeded the remaining two.

From all sides, especially from English, French, and Italians (Grandesso, Silvester, Clover, Chiene), came the assertion that long before my discovery this or that surgeon had adopted the means of binding the limbs to be amputated, or had raised them, and also that the practice of binding with india-rubber bands was an old story.

To this I agreed, not however without intimating that I had myself practiced and taught the method of binding and raising the condemned limbs, without however possessing the knowledge that not alone Brueninghausen, but surgeons of the sixteenth century, had practiced the same methods.

I was however in a position to argue that this was a matter not altogether confined to amputations, but that the importance of my proposition, the entirely novel, and as yet never propounded idea, was that we surgeons, whenever possible, should prevent the loss of blood, and that we are in a position to effect this by binding and tying so that the blood would be driven out exactly as we want it. One of our colleagues experienced an amusing deception. He volunteered the information at the following congress that bloodless operations had been in fact performed two hundred years ago, and had been commented upon by an anatomist named Bils. His experience had been explained by one Olans Barrichius under the title: *De Bilsii artificio incruentæ sectionis*, etc. (*fieri sanguine in aliam corporis partem compulso, ibiqui vinculis injectis retento*, etc.), and had furthermore been lauded by Albert von Haller in his Bibliotheca Anatomica, T. I., p. 495, 1774.

My surprise was expressed that the method of those surgeons had not been regarded, and was even entirely forgotten. Keeping the drift of those writings before me until I discovered and obtained the desired key of information from our University library, I then found that Bils had made a totally different discovery and description of it. He had, in order to show his pupils the branching of veins in the limbs, compressed the blood-vessels of animals as if for venesection, and then had exposed the enlarged or dilated vessels by means of the fingers and knife in a slow and careful manner, without injuring a single blood-vessel.

These claims gradually subsided; still at the same time opinions were advanced that showed the many dangerous disadvantages to which my discovery might lead. Even warnings were included, whilst my colleagues advised a discontinuance of such a process, and desired a return to the old method of the "tourniquet" or digital compression. Above all, it was asserted that injury to the compressed nerves, even decay of the edges of the wound often took place, and that on the whole it would be better to ignore the discovery altogether.

It has been my untiring effort to increase the practical effects of my discovery, and from time to time at our different congresses I have asserted that the disadvantages which were alleged against local anemia originated not in the procedure itself, but in mistaken practice. More especially does this lie in binding the rubber tube too tightly or in the too speedy removal of the bandage.

Nevertheless these reproaches drag themselves from one pamphlet to another, and from one edition to another, in opposition to "local anemia," and show that the authors have never taken the trouble to acquaint themselves with the improvements that have been added in the original discovery.

. . . . . . . . . . . . .

Expulsion of blood from one portion of the body to another portion has produced so many beneficial results that its advantages can scarcely be doubted any longer. Thus I live with the conviction that "local anemia" has the unquestioned right to be regarded as one of the most important adjuvants of practical surgery and that as such it will retain its influence.

---

DR. GONIN, of Lyons, sends a note to the Paris Academy of Medicine, recommending formol for mosquito and gnat bites; also for bites from small animals. The bites should be covered with formol by a small brush or the surface of the cork of the bottle containing it. After evaporation the formol is again applied. The soothing effect is instantaneous; there is never any inflammation. M. Gonin asserts that formol is efficacious for serpent and scorpion bites.—*British Medical Journal.*

